# Supplementary material for: Proton Therapy for Advanced Juvenile Nasopharyngeal Angiofibroma
Source: Cancers (Basel). 2023 Oct 17;15(20):5022. doi: 10.3390/cancers15205022 (PMC10605854; doi:10.3390/cancers15205022)
Supplement: Supplementary file 1 [file cancers-15-05022-s001.zip › cancers-2639159-supplementary.pdf]

Supplementary Tables

Table S1. Dosimetric parameters for organs at risk (OAR), extracted from the respective dose volume histogram of proton and photon plans. V_{xGy}: volume of the OAR receiving a minimum dose of x Gy; $D_{0.03cm3}$: minimum dose received in 0.03 cm^3 of the OAR; $D_{x\%}$: minimum dose received by x% of the OAR; D_{mean}: average dose; CTV: clinical target volume; ID: integral dose; IL: ipsilateral; CL: contralateral.

**Brain**	**$D_{0.03cm3}$, $D_{2\%}$, D_{mean}, V_{20Gy}, ID**
Brain (without CTV)	$D_{0.03cm3}$, $D_{2\%}$, $D_{50\%}$, D_{mean}, V_{10Gy}, V_{15Gy}, V_{20Gy}, V_{35Gy}, ID
Brain supratentorial	$D_{0.03cm3}$, $D_{2\%}$, $D_{50\%}$, D_{mean}, V_{10Gy}, V_{15Gy}, V_{20Gy}, ID
Brain infratentorial	$D_{0.03cm3}$, $D_{2\%}$, $D_{50\%}$, D_{mean}, V_{10Gy}, V_{15Gy}, V_{20Gy}, ID
Cerebellum	$D_{0.03cm3}$, $D_{2\%}$, $D_{50\%}$, D_{mean}, V_{10Gy}, V_{15Gy}, V_{20Gy}, ID
Cerebellum anterior	$D_{0.03cm3}$, $D_{2\%}$, $D_{50\%}$, D_{mean}, V_{10Gy}, V_{15Gy}, V_{20Gy}, ID
Cerebellum posterior	$D_{0.03cm3}$, $D_{2\%}$, $D_{50\%}$, D_{mean}, V_{10Gy}, V_{15Gy}, V_{20Gy}, ID
Brainstem	$D_{0.03cm3}$, $D_{2\%}$, D_{mean}, ID
Hippocampus bilateral	$D_{0.03cm3}$, $D_{2\%}$, $D_{40\%}$, $D_{50\%}$, D_{mean}, ID
Hippocampus IL	$D_{0.03cm3}$, $D_{2\%}$, $D_{50\%}$, D_{mean}, ID
Hippocampus CL	$D_{0.03cm3}$, $D_{2\%}$, $D_{50\%}$, D_{mean}, ID
Pituitary	$D_{0.03cm3}$, $D_{2\%}$, D_{mean}, ID
Chiasma	$D_{0.03cm3}$, $D_{2\%}$, D_{mean}, ID
Optic nerve IL	$D_{0.03cm3}$, $D_{2\%}$, D_{mean}, ID
Optic nerve CL	$D_{0.03cm3}$, $D_{2\%}$, D_{mean}, ID
Eye IL	$D_{0.03cm3}$, $D_{2\%}$, D_{mean}, V_{10Gy}, ID
Eye CL	$D_{0.03cm3}$, $D_{2\%}$, D_{mean}, V_{10Gy}, ID
Lens IL	$D_{0.03cm3}$, $D_{2\%}$, D_{mean}, ID
Lens CL	$D_{0.03cm3}$, $D_{2\%}$, D_{mean}, ID
Lacrimal gland IL	$D_{0.03cm3}$, $D_{2\%}$, D_{mean}, ID
Lacrimal gland CL	$D_{0.03cm3}$, $D_{2\%}$, D_{mean}, ID
Cochlea IL	$D_{0.03cm3}$, $D_{2\%}$, D_{mean}, ID
Cochlea CL	$D_{0.03cm3}$, $D_{2\%}$, D_{mean}, ID
Parotis IL	$D_{0.03cm3}$, $D_{2\%}$, D_{mean}, ID
Parotis CL	$D_{0.03cm3}$, $D_{2\%}$, D_{mean}, ID
Skin	$D_{0.03cm3}$, $D_{2\%}$, $D_{5\%}$, $D_{50\%}$, D_{mean}, V_{10Gy}, V_{15Gy}, V_{20Gy}, V_{35Gy}, ID

Table S2. Dosimetric parameters regarding CTV. Doses are expressed in percentage of the prescribed dose. Volumes are expressed in percentage of the CTV volume. Relative values are expressed in %; CTV: clinical target volume; V_{xGy}: volume of the CTV receiving a minimum dose of x Gy; $D_{0.03cm3}$: minimum dose received in 0.03 cm^3 of the CTV; Dx%: minimum dose received by x% of the CTV; HI: homogeneity index; CI: conformity Index; SD: standard deviation. Δabs and Δrel: difference in absolute and relative values regarding a specific dosimetric parameter between proton and photon plans, respectively.

		Proton			Photon			Δ_{abs}(Proton - Photon)			Δ_{rel}(Proton - Photon)			
		Mean		SD	Mean		SD	Mean		SD	Mean		SD	p-value
CTV	D0.03cm³	105.5	±	2.3	104.4	±	0.7	1.1	±	2.2	1.0	±	2.1	0.193
	D2%	102.9	±	0.7	103.1	±	0.4	-0.2	±	0.7	-0.2	±	0.7	0.263
	D5%	102.2	±	0.5	102.7	±	0.4	-0.5	±	0.5	-0.5	±	0.6	**0.047**
	D95%	97.1	±	1.8	97.3	±	0.6	-0.2	±	0.8	-0.2	±	1.9	0.769
	D98%	95.9	±	2.4	96.7	±	0.8	-0.8	±	2.3	-0.8	±	2.4	0.568
	V95%	97.8	±	4.4	99.6	±	0.5	-1.8	±	4.3	-1.8	±	4.3	0.250
	V105%	0.2	±	0.4	0.1	±	0.1	0.2	±	0.4	-	±	-	-
	V107%	0.1	±	0.2	0.0	±	0.0	0.1	±	0.2	-	±	-	-
	HI	5.1	±	1.8	5.4	±	0.8	-0.3	±	1.8	-5.6	±	30.5	0.695
	CI	0.58	±	0.18	0.56	±	0.16	0.03	±	0.06	4.8	±	9.0	0.287

Table S3. Dosimetric parameters related to organs at risk between proton and photon plans. Relative values are expressed in %. Significant results are highlighted in bold. CTV: clinical target volume; CL: contralateral; IL: ipsilateral, SD: standard deviation; Δ_{abs} and Δ_{rel}: difference in absolute and relative values regarding a specific dosimetric parameter between proton and photon plans, respectively.

		Proton				Photon			Δ_{abs}(Proton - Photon)			Δ_{rel}(Proton - Photon)			
		Mean	±	SD		Mean	±	SD	Mean	±	SD	Mean	±	SD	p-value
Brain	D0.03cm³ (Gy)	46.0	±	0.7		46.3	±	0.5	-0.3	±	0.5	-0.6	±	1.0	0.074
	D2% (Gy)	31.7	±	11.6		34.6	±	8.6	-3.0	±	4.0	-11.1	±	15.1	**0.027**
	Dmean (Gy)	3.2	±	1.6		5.3	±	1.7	-2.1	±	1.6	-37.7	±	29.1	**0.006**
	V20Gy (cm³)	87.4	±	57.2		119.8	±	68.3	-32.4	±	24.0	-29.4	±	19.0	**0.004**
	V20Gy (%)	5.0	±	3.0		7.2	±	3.6	-2.0	±	1.4				
	ID (Gy.cm³)	5364.0	±	2775.3		8734.3	±	3393.3	-3370.3	±	2755.6				
Brain (without CTV)	D0.03cm³ (Gy)	45.4	±	0.9		45.9	±	0.6	-0.4	±	0.6	-1.0	±	1.4	**0.006**
	D2% (Gy)	29.7	±	10.0		32.9	±	7.0	-3.2	±	3.8	-12.0	±	14.6	**0.004**
	D50% (Gy)	0.1	±	0.1		1.4	±	1.3	-1.4	±	1.3	-93.3	±	12.0	**0.002**
	Dmean (Gy)	3.0	±	1.4		5.1	±	1.6	-2.1	±	1.7	-39.1	±	29.3	**0.006**
	V10Gy (cm³)	186.4	±	111.7		285.7	±	110.1	-99.3	±	129.4	-31.3	±	43.9	**0.049**
	V15Gy (cm³)	118.4	±	68.8		163.7	±	74.9	-45.2	±	50.2	-28.3	±	29.1	**0.020**
	V20Gy (cm³)	77.6	±	46.7		110.0	±	57.4	-32.4	±	24.1	-30.9	±	18.9	**0.004**
	V35Gy (cm³)	28.2	±	20.4		33.9	±	22.9	-5.7	±	3.8	-20.0	±	14.7	**0.002**
	V10Gy (%)	11.4	±	6.7		17.6	±	6.3	-6.1	±	7.9				
	V15Gy (%)	7.2	±	4.0		10.0	±	4.1	-2.8	±	3.0				
	V20Gy (%)	4.7	±	2.6		6.7	±	3.2	-2.0	±	1.4				
	V35Gy (%)	1.7	±	1.2		2.1	±	1.4	-0.3	±	0.2				
	ID (Gy.cm³)	4924.2	±	2432.6		8294.5	±	2969.2	-3370.3	±	2755.4				
Brain supratentorial	D0.03cm³ (Gy)	46.0	±	0.7		46.2	±	0.4	-0.3	±	0.4	-0.6	±	1.0	0.111
	D2% (Gy)	31.5	±	11.7		34.8	±	8.5	-3.3	±	3.9	-12.0	±	14.9	**0.027**
	D50% (Gy)	0.1	±	0.1		0.9	±	0.5	-0.9	±	0.6	-88.2	±	24.0	**0.002**
	Dmean (Gy)	3.3	±	1.7		4.2	±	1.8	-0.9	±	1.9	-15.3	±	47.1	0.193
	V10Gy (cm³)	175.8	±	110.3		171.9	±	84.8	3.9	±	115.0	14.7	±	84.4	0.770
	V15Gy (cm³)	112.5	±	70.2		130.0	±	66.4	-17.5	±	46.5	-13.3	±	38.9	0.232
	V20Gy (cm³)	74.9	±	50.3		100.4	±	56.9	-25.4	±	22.2	-27.5	±	21.7	**0.010**
	V10Gy (%)	12.5	±	7.7		12.1	±	5.3	0.4	±	8.3				
	V15Gy (%)	7.9	±	4.5		9.2	±	4.2	-1.3	±	3.3				
	V20Gy (%)	5.2	±	3.1		7.1	±	3.6	-1.8	±	1.6				
	ID (Gy.cm³)	4692.0	±	2507.7		5982.1	±	2883.4	-1290.1	±	2608.2				
Brain infratentorial	D0.03cm³ (Gy)	43.1	±	6.8		44.1	±	5.5	-1.0	±	1.6	-2.8	±	4.9	**0.035**
	D2% (Gy)	31.5	±	12.5		32.1	±	8.9	-0.6	±	4.4	-5.5	±	20.3	0.492
	D50% (Gy)	0.1	±	0.0		10.1	±	1.7	-10.0	±	1.7	-99.3	±	0.4	**0.002**
	Dmean (Gy)	2.8	±	1.4		11.4	±	2.1	-8.7	±	1.3	-76.7	±	8.9	**0.002**
	V10Gy (cm³)	20.6	±	10.9		124.9	±	38.6	-104.3	±	32.5	-83.6	±	6.3	**0.002**
	V15Gy (cm³)	15.9	±	9.4		43.9	±	21.6	-28.0	±	13.6	-65.2	±	12.2	**0.002**
	V20Gy (cm³)	12.6	±	8.1		19.6	±	13.4	-7.0	±	6.3	-38.6	±	21.7	**0.002**
	V10Gy (%)	8.4	±	4.4		52.2	±	17.2	-43.8	±	15.6				
	V15Gy (%)	6.4	±	3.9		17.9	±	7.7	-11.4	±	4.7				
	V20Gy (%)	5.1	±	3.3		7.8	±	4.9	-2.7	±	2.0				
	ID (Gy.cm³)	679.0	±	353.5		2770.8	±	653.4	-2091.8	±	383.0				
Cerebellum	D0.03cm³ (Gy)	13.7	±	10.6	±	26.3	±	7.4	-12.6	±	5.3	-53.5	±	28.6	**0.002**
	D2% (Gy)	3.1	±	2.9	±	18.8	±	4.1	-15.8	±	2.3	-85.4	±	10.9	**0.002**
	D50% (Gy)	0.0	±	0.0	±	9.6	±	1.6	-9.6	±	1.6	-99.7	±	0.2	**0.002**
	Dmean (Gy)	0.3	±	0.2	±	10.0	±	1.8	-9.7	±	1.6	-97.3	±	1.8	**0.002**
	V10Gy (cm³)	0.5	±	0.9	±	69.3	±	28.2	-68.7	±	27.8	-99.4	±	1.0	**0.002**
	V15Gy (cm³)	0.2	±	0.4	±	13.4	±	10.8	-13.2	±	10.7	-98.6	±	1.8	**0.002**
	V20Gy (cm³)	0.1	±	0.2	±	3.1	±	4.1	-3.0	±	4.1	-66.6	±	46.2	**0.016**
	V10Gy (%)	0.4	±	0.6		45.9	±	19.9	-45.5	±	19.6				
	V15Gy (%)	0.2	±	0.3		8.6	±	6.3	-8.5	±	6.1				
	V20Gy (%)	0.1	±	0.1		1.9	±	2.3	-1.8	±	2.3				
	ID (Gy.cm³)	43.6	±	31.2		1522.7	±	312.4	-1479.1	±	295.7				
Cerebellum anterior	D0.03cm³ (Gy)	9.2	±	9.3		20.8	±	6.9	-11.5	±	5.8	-61.4	±	30.1	**0.002**
	D2% (Gy)	3.7	±	4.2		15.8	±	4.8	-12.1	±	3.5	-79.4	±	17.9	**0.002**
	D50% (Gy)	0.1	±	0.1		7.1	±	2.5	-7.0	±	2.5	-98.9	±	0.9	**0.002**
	Dmean (Gy)	0.4	±	0.4		7.5	±	2.4	-7.1	±	2.2	-95.2	±	4.0	**0.002**
	V10Gy (cm³)	0.1	±	0.1		4.3	±	3.8	-4.2	±	3.7	-98.5	±	2.5	**0.002**
	V15Gy (cm³)	0.0	±	0.1		0.6	±	0.6	-0.5	±	0.6	-70.9	±	40.5	**0.016**
	V20Gy (cm³)	0.0	±	0.0		0.2	±	0.3	-0.2	±	0.3	-53.1	±	46.7	**0.031**
	V10Gy (%)	0.5	±	1.2		25.7	±	23.1	-25.2	±	22.7				
	V15Gy (%)	0.2	±	0.6		3.2	±	3.2	-2.9	±	2.9				
	V20Gy (%)	0.1	±	0.3		1.0	±	1.6	-0.9	±	1.4				
	ID (Gy.cm³)	6.2	±	5.2		128.1	±	39.3	-121.9	±	38.2				

Table S3 (continued):

Cerebellum posterior	D0.03cm^3 (Gy)	12.1	±	9.5	26.0	±	7.4	-13.9	±	4.2	-58.5	±	25.1	**0.002**
	D2% (Gy)	3.0	±	3.1	19.0	±	4.2	-16.0	±	2.2	-86.0	±	11.3	**0.002**
	D50% (Gy)	0.0	±	0.0	9.9	±	1.6	-9.8	±	1.6	-99.8	±	0.2	**0.002**
	Dmean (Gy)	0.3	±	0.2	10.3	±	1.7	-10.0	±	1.5	-97.5	±	1.9	**0.002**
	V10Gy (cm^3)	0.4	±	0.8	65.0	±	25.4	-64.6	±	25.1	-99.4	±	1.0	**0.002**
	V15Gy (cm^3)	0.2	±	0.4	12.8	±	10.3	-12.6	±	10.2	-98.8	±	1.9	**0.002**
	V20Gy (cm^3)	0.1	±	0.2	2.9	±	3.9	-2.8	±	3.8	-67.2	±	46.6	**0.016**
	V10Gy (%)	0.3	±	0.7	48.4	±	19.8	-48.0	±	19.5				
	V15Gy (%)	0.1	±	0.3	9.3	±	6.9	-9.2	±	6.7				
	V20Gy (%)	0.1	±	0.1	2.0	±	2.5	-2.0	±	2.4				
	ID (Gy.cm^3)	37.2	±	28.3	1395.7	±	285.2	-1358.6	±	271.9				
Brainstem	D0.03cm^3 (Gy)	36.5	±	8.8	37.2	±	8.0	-0.7	±	3.0	-2.1	±	10.3	0.770
	D2% (Gy)	31.1	±	11.0	31.4	±	9.0	-0.2	±	3.2	-2.3	±	12.9	0.625
	Dmean (Gy)	8.2	±	4.8	14.5	±	3.5	-6.3	±	2.0	-47.3	±	20.8	**0.002**
	ID (Gy.cm^3)	226.5	±	137.2	397.3	±	124.2	-170.8	±	53.6				
Hippocampus (bilateral)	D0.03cm^3 (Gy)	30.2	±	11.1	34.5	±	6.2	-4.3	±	5.4	-15.3	±	20.5	**0.049**
	D2% (Gy)	27.7	±	11.3	32.0	±	7.0	-4.3	±	5.1	-16.8	±	21.7	**0.049**
	D40% (Gy)	4.3	±	5.5	13.7	±	8.1	-9.3	±	5.1	-70.4	±	24.3	**0.002**
	D50% (Gy)	2.3	±	3.6	10.3	±	6.9	-8.0	±	4.8	-80.0	±	17.1	**0.002**
	Dmean (Gy)	6.0	±	3.9	12.2	±	5.4	-6.2	±	3.6	-49.8	±	27.1	**0.004**
	ID (Gy.cm^3)	24.5	±	14.5	51.7	±	24.7	-27.3	±	18.3				
Hippocampus CL	D0.03cm^3 (Gy)	20.1	±	15.5	28.9	±	11.0	-8.9	±	7.5	-39.5	±	31.3	**0.006**
	D2% (Gy)	19.1	±	15.0	28.2	±	11.0	-9.1	±	7.3	-41.0	±	31.1	**0.006**
	D50% (Gy)	1.5	±	2.3	9.6	±	6.3	-8.1	±	4.9	-86.5	±	12.6	**0.002**
	Dmean (Gy)	4.0	±	4.0	11.4	±	5.6	-7.4	±	3.7	-69.6	±	20.0	**0.002**
	ID (Gy.cm^3)	7.9	±	7.3	24.1	±	13.2	-16.2	±	10.2				
Hippocampus IL	D0.03cm^3 (Gy)	29.3	±	11.8	33.4	±	6.7	-4.1	±	5.5	-15.7	±	22.4	**0.049**
	D2% (Gy)	28.6	±	11.9	32.7	±	6.9	-4.1	±	5.4	-16.3	±	23.0	**0.049**
	D50% (Gy)	5.5	±	6.2	11.5	±	7.6	-6.0	±	6.4	-35.0	±	112.2	**0.037**
	Dmean (Gy)	8.1	±	4.9	13.2	±	5.3	-5.0	±	4.1	-38.2	±	37.6	**0.010**
	ID (Gy.cm^3)	16.6	±	9.3	27.7	±	12.3	-11.1	±	9.1				
Pituitary	D0.03cm^3 (Gy)	40.9	±	6.8	42.9	±	4.0	-2.0	±	4.1	-5.1	±	10.1	**0.049**
	D2% (Gy)	41.9	±	5.9	43.5	±	3.4	-1.6	±	3.7	-4.0	±	9.0	0.064
	Dmean (Gy)	37.7	±	10.6	40.3	±	6.5	-2.6	±	4.5	-8.4	±	14.7	0.131
	ID (Gy.cm^3)	15.3	±	7.6	16.6	±	7.4	-1.3	±	3.0				
Chiasma	D0.03cm^3 (Gy)	37.5	±	11.6	38.6	±	11.5	-1.1	±	2.9	-1.6	±	13.6	0.139
	D2% (Gy)	37.8	±	10.5	39.1	±	11.0	-1.4	±	3.3	-1.0	±	15.9	0.160
	Dmean (Gy)	27.2	±	14.9	28.3	±	13.6	-1.1	±	5.1	-5.6	±	26.9	0.695
	ID (Gy.cm^3)	43.2	±	29.3	42.7	±	25.6	0.5	±	9.3				
Optic nerve CL	D0.03cm^3 (Gy)	36.8	±	10.9	40.6	±	6.3	-3.8	±	6.3	-10.9	±	17.7	0.064
	D2% (Gy)	37.2	±	10.4	41.0	±	5.8	-3.8	±	6.1	-10.7	±	16.9	**0.027**
	Dmean (Gy)	28.2	±	12.9	30.4	±	9.1	-2.2	±	4.8	-11.8	±	22.2	0.432
	ID (Gy.cm^3)	33.6	±	33.3	36.1	±	32.6	-2.6	±	5.2				
Optic nerve IL	D0.03cm^3 (Gy)	42.3	±	2.8	43.4	±	1.8	-1.1	±	1.8	-2.6	±	4.2	0.084
	D2% (Gy)	42.7	±	2.5	43.9	±	1.5	-1.1	±	1.8	-2.6	±	4.2	0.064
	Dmean (Gy)	35.5	±	8.5	35.7	±	7.9	-0.2	±	2.0	-0.6	±	7.5	0.922
	ID (Gy.cm^3)	37.6	±	28.0	38.2	±	28.5	-0.5	±	2.0				
Eye CL	D0.03cm^3 (Gy)	20.0	±	8.9	19.0	±	7.9	1.0	±	4.2	4.5	±	23.3	0.770
	D2% (Gy)	17.2	±	8.4	15.9	±	6.8	1.3	±	4.2	6.8	±	29.1	0.557
	Dmean (Gy)	6.4	±	4.4	6.3	±	1.8	0.1	±	3.4	-3.5	±	51.4	0.770
	V10Gy (cm^3)	2.0	±	2.3	1.3	±	1.2	0.6	±	1.8	35.6	±	114.5	0.322
	V10Gy (%)	21.0	±	23.8	14.5	±	12.7	6.5	±	18.9	35.6	±	114.5	
	ID (Gy.cm^3)	59.4	±	42.2	57.4	±	18.2	2.0	±	31.8				
Eye IL	D0.03cm^3 (Gy)	28.2	±	11.8	31.4	±	11.5	-3.2	±	2.6	-11.9	±	10.2	**0.002**
	D2% (Gy)	25.6	±	12.1	28.2	±	11.2	-2.5	±	2.1	-12.2	±	12.8	**0.002**
	Dmean (Gy)	11.1	±	7.6	10.1	±	6.2	0.9	±	2.6	0.9	±	36.3	0.311
	V10Gy (cm^3)	3.9	±	3.0	3.2	±	2.5	0.7	±	1.2	2.5	±	55.5	0.232
	V10Gy (%)	42.0	±	31.6	34.5	±	26.2	7.5	±	12.6				
	ID (Gy.cm^3)	102.8	±	73.1	93.7	±	61.1	9.1	±	23.8				
Lens CL	D0.03cm^3 (Gy)	4.4	±	3.7	4.0	±	0.8	0.4	±	3.4	6.6	±	76.0	0.922
	D2% (Gy)	5.3	±	4.5	4.8	±	1.0	0.5	±	4.2	7.9	±	81.0	0.846
	Dmean (Gy)	3.4	±	2.9	3.4	±	0.6	0.0	±	2.8	-0.4	±	73.7	0.846
	ID (Gy.cm^3)	0.7	±	0.6	0.7	±	0.2	0.0	±	0.6				
Lens IL	D0.03cm^3 (Gy)	7.0	±	7.7	6.6	±	8.2	0.4	±	2.8	18.2	±	77.5	0.846
	D2% (Gy)	8.3	±	8.7	7.8	±	9.1	0.5	±	3.0	14.1	±	60.1	0.695
	Dmean (Gy)	5.3	±	5.7	5.1	±	6.5	0.2	±	2.7	19.0	±	78.2	0.922
	ID (Gy.cm^3)	1.2	±	1.4	1.2	±	1.7	0.0	±	0.5				

Table S3 (continued):

Lacrimal gland CL	D0.03cm³ (Gy)	5.6	±	5.0	9.6	±	3.0	-4.0	±	5.8	-38.0	±	49.6	0.064
	D2% (Gy)	5.9	±	4.9	10.2	±	3.0	-4.2	±	6.0	-37.3	±	49.9	**0.049**
	Dmean (Gy)	3.2	±	3.8	5.1	±	1.9	-1.9	±	4.0	-37.1	±	61.6	0.105
	ID (Gy.cm³)	3.5	±	5.8	5.0	±	2.4	-1.5	±					
Lacrimal gland IL	D0.03cm³ (Gy)	13.0	±	10.9	14.0	±	8.7	-1.0	±	5.2	-7.4	±	54.3	0.846
	D2% (Gy)	13.8	±	11.3	15.5	±	8.9	-1.7	±	5.1	-13.8	±	48.1	0.557
	Dmean (Gy)	7.0	±	6.6	7.2	±	5.7	-0.3	±	2.8	-4.7	±	59.5	0.922
	ID (Gy.cm³)	6.4	±	7.7	6.9	±	7.2	-0.6	±	2.3				
Cochlea CL	D0.03cm³ (Gy)	8.3	±	7.0	22.1	±	4.7	-13.8	±	6.3	-63.2	±	29.0	**0.002**
	D2% (Gy)	9.8	±	7.4	23.7	±	4.9	-14.0	±	6.6	-59.6	±	28.1	**0.002**
	Dmean (Gy)	7.5	±	6.4	21.1	±	4.5	-13.6	±	5.8	-65.3	±	28.1	**0.002**
	ID (Gy.cm³)	0.9	±	0.8	2.6	±	1.1	-1.7	±	0.9				
Cochlea IL	D0.03cm³ (Gy)	24.0	±	13.7	29.9	±	5.3	-5.9	±	9.4	-24.1	±	35.6	0.232
	D2% (Gy)	28.0	±	13.5	32.5	±	5.8	-4.6	±	9.2	-17.4	±	31.8	0.322
	Dmean (Gy)	21.8	±	12.4	28.1	±	4.5	-6.3	±	8.9	-26.2	±	35.6	0.102
	ID (Gy.cm³)	3.0	±	2.7	3.7	±	1.9	-0.7	±	1.3				
Parotis CL	D0.03cm³ (Gy)	15.9	±	6.8	18.8	±	5.3	-2.9	±	5.6	-13.6	±	34.7	0.193
	D2% (Gy)	14.1	±	6.0	16.6	±	4.3	-2.6	±	5.5	-13.1	±	37.8	0.160
	Dmean (Gy)	6.6	±	4.3	8.3	±	2.8	-1.6	±	3.0	-21.8	±	31.8	0.064
	ID (Gy.cm³)	109.6	±	82.5	134.6	±	62.0	-25.1	±	49.2				
Parotis IL	D0.03cm³ (Gy)	27.4	±	12.4	29.6	±	9.8	-2.1	±	5.2	-9.6	±	21.9	0.275
	D2% (Gy)	23.7	±	12.8	26.4	±	9.9	-2.8	±	6.1	-13.9	±	28.5	0.232
	Dmean (Gy)	10.8	±	9.3	11.3	±	5.2	-0.5	±	4.9	-15.7	±	44.5	0.695
	ID (Gy.cm³)	153.9	±	128.0	161.2	±	72.7	-7.3	±	70.1				
Skin	D0.03cm³ (Gy)	34.7	±	9.5	36.3	±	9.4	-1.5	±	2.9	-4.0	±	10.8	0.193
	D2% (Gy)	20.9	±	6.9	23.2	±	6.2	-2.3	±	2.7	-10.7	±	12.2	**0.049**
	D5% (Gy)	14.2	±	5.3	18.0	±	4.3	-3.8	±	2.0	-23.2	±	13.7	**0.002**
	Dmean (Gy)	2.3	±	1.1	4.4	±	1.4	-2.1	±	0.5	-50.0	±	9.1	**0.002**
	V10Gy (cm³)	52.4	±	30.9	95.8	±	44.1	-43.3	±	17.8	-47.4	±	12.3	**0.002**
	V15Gy (cm³)	29.5	±	27.0	45.3	±	27.9	-15.9	±	7.4	-42.3	±	18.6	**0.002**
	V20Gy (cm³)	14.2	±	13.0	21.0	±	16.6	-6.8	±	6.5	-28.7	±	50.4	**0.004**
	V35Gy (cm³)	1.8	±	3.2	2.0	±	2.9	-0.2	±	0.8	-20.0	±	29.8	0.438
	ID (Gy.cm³)	1294.0	±	625.4	2498.2	±	876.4	-1204.3	±	311.8				

Table S4. Estimated intelligence quotient (IQ) following radiotherapy, normal tissue complication probabilities (NTCP) for all organs at risk, and risk ratio (RR) for secondary CNS malignancies. NTCP are expressed in %, IQ (*) is expressed in points. Significant results are highlighted in bold. CTV: clinical target volume; CL: contralateral; IL: ipsilateral; SD: standard deviation; GH: growth hormone; HT: hypothyroidism; ACTH: adrenocorticotropic hormone; Δ_{abs}: difference in absolute values regarding a specific complication between proton and photon plans.

		Proton			Photon			Δ_{abs}(Proton - Photon)			
Organ at risk	Complication	Mean	±	SD	Mean	±	SD	Mean	±	SD	p-value
Brain	IQ (Merchant et al. 2006)*	115.6	±	4.9	114.4	±	5.1	1.2	±	0.9	**0.006**
Brain	IQ (Mahajan et al. 2021)*	107.6	±	0.3	107.2	±	0.5	0.4	±	0.4	**0.006**
Brain	Neurocognitive impairment (IQ<85)	1.9	±	1.4	2.2	±	1.6	-0.4	±	0.2	**0.002**
Brain	Necrosis	1.1	±	0.1	1.2	±	0.1	0.0	±	0.1	0.061
Infratentorial Brain	IQ*	112.4	±	4.1	107.6	±	4.3	4.7	±	0.7	**0.002**
Supratentorial brain	IQ*	121.3	±	5.9	120.9	±	6.0	0.4	±	0.9	0.193
Brain (without CTV)	Necrosis	0.0	±	0.0	0.0	±	0.0	0.0	±	0.0	0.063
Brainstem	Necrosis	0.0	±	0.0	0.0	±	0.0	0.0	±	0.0	0.500
Chiasma	Blindness	0.3	±	0.5	0.3	±	0.4	0.0	±	0.1	0.961
Optic nerve CL	Blindness	0.2	±	0.3	0.2	±	0.2	0.0	±	0.1	0.715
Optic nerve IL	Blindness	0.4	±	0.4	0.3	±	0.3	0.0	±	0.1	0.846
Cochlea CL	Tinnitus	0.8	±	0.7	3.7	±	2.1	-2.9	±	1.8	**0.002**
Cochlea CL	Hearing loss	0.0	±	0.0	0.0	±	0.0	0.0	±	0.0	1.000
Cochlea IL	Tinnitus	7.6	±	7.7	8.8	±	4.6	-1.1	±	4.2	0.432
Cochlea IL	Hearing loss	0.0	±	0.1	0.0	±	0.0	0.0	±	0.1	0.703
Hippocampus (bilateral)	Delayed recall	8.5	±	10.7	28.3	±	24.1	-19.9	±	15.6	**0.002**
Pituitary	Endocrine dysfunction	1.4	±	1.1	1.5	±	1.1	-0.1	±	0.5	0.770
Pituitary	GH-deficiency	62.6	±	20.1	67.2	±	13.1	-4.6	±	7.7	0.131
Pituitary	Hypothyroidism	43.9	±	18.6	47.4	±	13.7	-3.5	±	5.6	0.131
Pituitary	ACTH-deficiency	25.0	±	9.2	26.7	±	6.9	-1.7	±	2.7	0.131
Lacrimal gland IL	Ocular toxicity	18.7	±	28.4	17.2	±	24.7	1.5	±	7.5	0.557
Lens CL	Cataract	0.7	±	1.4	0.2	±	0.1	0.6	±	1.4	1.000
Lens IL	Cataract	8.1	±	24.0	9.3	±	29.0	-1.2	±	5.0	0.492
Parotis CL	Xerostomia	2.2	±	1.6	2.5	±	1.0	-0.3	±	0.9	0.084
Parotis IL	Xerostomia	5.9	±	7.9	4.4	±	3.8	1.5	±	4.6	0.695
Skin	Alopecia	45.3	±	10.3	52.7	±	8.4	-7.4	±	3.9	**0.002**
Skin	Erythema	19.4	±	3.2	19.6	±	2.9	-0.2	±	0.8	0.688

		RR (Photon/Proton)			
		Mean	±	SD	p-value
Brain	Secondary malignancies	2.1	±	1.2	**0.006**
